# Newly arrived migrants did not represent an additional COVID-19 burden for Italy: data from the italian information flow

**DOI:** 10.1186/s12992-023-00926-9

**Published:** 2023-05-02

**Authors:** Leuconoe Grazia Sisti, Anteo Di Napoli, Alessio Petrelli, Alessandra Diodati, Andrea Cavani, Concetta Mirisola, Gianfranco Costanzo

**Affiliations:** 1 Health Directorate, National Institute for Health, Migration and Poverty (INMP), Via di San Gallicano, 25/a, 00153 Roma, Italy; 2 Center for Global Health Research and Studies, Department Life Sciences and Public Health, Università Cattolica del Sacro Cuore, L.go F. Vito, 1, 00168 Roma, Italy; 3 Epidemiology Unit, National Institute for Health, Migration and Poverty (INMP), Via di San Gallicano, 25/a, 00153 Roma, Italy; 4 Global Health and Health Cooperation Unit, National Institute for Health, Migration and Poverty (INMP), Via di San Gallicano, 25/a, 00153 Roma, Italy; 5 Scientific Directorate, National Institute for Health, Migration and Poverty (INMP), Via di San Gallicano, 25/a, 00153 Roma, Italy; 6 Directorate General, National Institute for Health, Migration and Poverty (INMP), Via di San Gallicano, 25/a, 00153 Roma, Italy

**Keywords:** COVID-19, Migrant health, Public health

## Abstract

**Background:**

During the COVID-19 pandemic, migrants arriving in host countries irregularly have not infrequently been perceived as increasing the COVID-19 burden. Italy is a transit and destination country for migrants who cross the Central Mediterranean route and, during the pandemic, all migrants who landed on Italian shores were COVID-19 tested and quarantined. Our study aimed to investigate the impact of the SARS-CoV-2 infection among migrants who landed on the Italian coasts by analyzing both incidence and health outcomes.

**Methods:**

A retrospective observational study has been designed. The population of interest was represented by 70,512 migrants (91% male, 99% <60 years old) who landed in Italy between January 2021 and 2022. SARS-CoV-2 incidence rate per 1,000 (with 95%CI) in migrants and the resident population in Italy of the corresponding age group was computed. The incidence rate ratio (IRR) was used to compare the incidence rates in migrants and the resident population.

**Results:**

2,861 migrants out of those landed in Italy during the observation period tested positive, with an incidence rate of 40.6 (39.1–42.1) cases per 1,000. During the same period, 177.6 (177.5-177.8) cases per 1,000 were reported in the resident population, with an IRR of 0.23 (0.22–0.24). 89.7% of cases were male and 54.6% belonged to the 20–29 age group. 99% of cases reported no symptoms, no relevant comorbidities were reported and no cases were hospitalized.

**Conclusions:**

Our study found a low rate of SARS-CoV-2 infection in migrants reaching Italy by sea with an incidence rate that is roughly a quarter of that of the resident population. Thus, irregular migrants who arrived in Italy during the observation period did not increase the COVID-19 burden. Further studies are needed to investigate possible reasons for the low incidence observed in this population.

## Background

On March 11, 2020, the World Health Organization (WHO) declared the coronavirus disease 2019 (COVID-19) outbreak a global pandemic. The first pandemic wave strongly impacted China, Europe (in particular Italy, France, United Kingdom, and Germany) and the United States (US) and, despite an increase in cases observed in the following waves, Asia, except for India (about 43.2 million cases to date), and Africa (11.6 million cases) have been less impacted by COVID-19.

The pandemic has been characterized by border closure policies enacted to limit the spread of the virus potentially coming from outside the country.

In this scenario, as witnessed by similar situations in the past, migrants who have reached new countries irregularly, i.e. without complying with administrative procedures for legal entry, have not infrequently been perceived as posing a risk of an increased COVID-19 burden, [4], even when their countries of origin reported a low incidence of COVID-19. This concern was often justified by the possible under-reporting of cases in migrants’ countries of origin, especially in countries with limited economic resources.

Italy is a relevant transit and destination country for migrants entering irregularly. Most of them embark in Libya, Tunisia but also Algeria, cross the Mediterranean Route and land mainly along the coasts of Sicily, Calabria and Apulia. Once arrived in Italy, they are hosted in a multi-level reception system for migrants, managed by the Department for civil liberties and immigration of the Italian Ministry of Interior.

The COVID-19 pandemic did not stop the influx of migrants to Italy, which numbered 11,471 in 2019, 34,154 in 2020 and 67,040 in 2021 [5], and required the Italian government to apply the same public health measures to these people as it did to all foreign nationals who entered Italy during the pandemic. In particular, in April 2020, a circular of the Ministry of the Interior [6] expressly provided for COVID-19 fiduciary isolation for all newly arrived migrants, and the Italian Civil Protection Department issued guidance on the management of isolation of migrants rescued at sea or arriving on national territory as a result of autonomous landings, [7] (i.e. landings not intercepted at sea by Italian navy or coast guard or non-governmental organizations), also considering the use of dedicated boats, in addition to land-based facilities, to carry out fiduciary public health measures.

In July 2020, the Italian National Institute for Health, Migration, and Poverty (INMP), in line with its institutional mandate and in collaboration with other institutional bodies, published the “Interim operating procedures for the management of facilities with persons who are highly vulnerable and at high risk of health and social care exclusion during the COVID-19 epidemic” [8], further detailing recommendations for public health preventive measures and health surveillance in case of new arrivals of migrants.

The operating procedures, validated by the Italian Scientific Technical Committee in support of the response to the COVID-19 emergency and adopted by the Ministry of the Interior, recommended to perform - along with the assessment of any vulnerability or specific needs - a severe acute respiratory syndrome coronavirus 2 (SARS-CoV-2) nasopharyngeal swab to all newly arrived migrants detected at the border, whether land or maritime.

According to the result of the test, carried out by the staff of the local health authorities of the points of entry, newly arrived migrants were separated into different cohorts to accomplish proper prevention measures (isolation for positive cases, quarantine for high-risk exposure contacts, and distinct quarantine for low-risk exposure contacts) that followed the regulations currently in force, and were implemented in different facilities/ships or different areas of the same facilities.

In case of emergence of a positive case during the quarantine period, close contacts were actively surveilled with serial testing to timely identify and isolate secondary cases from the initial cohort. At the end of the period of isolation and quarantine, after a negative SARS-CoV-2 test, migrants were relocated to the ordinary facilities of the reception system for migrants.

To track positive cases detected both at the borders and in the Italian reception system for migrants, in January 2021 the Italian Ministry of Interior requested INMP to set up and manage a specific information flow providing timely information on SARS-CoV-2 infection incidence and health outcomes in both newly arrived migrants and those already hosted in the Italian reception system.

In this framework, our study is aimed at investigating the impact of the SARS-CoV-2 infection, in terms of both incidence and health outcomes, among migrants who landed on Italian coasts during the first year of information flow operation.

## Methods

### Study design, population and period of observation

A retrospective observational study has been designed. The population of interest was represented by all 70,512 [5] migrants (67,477 in 2021 and 3,035 in January 2022) irregularly arrived on the Italian coasts during the period of observation (from 4 January 2021 to 31 January 2022) and hosted in dedicated isolation and quarantine land-based and ship facilities after being all COVID-19 tested and before entering the Italian reception system for migrants. Regarding the demographic characteristics of the population, 80.71% were adults (91.04% males and 8.96% females, 99% under 60 years old) and 19.3% minors (unspecified in sex).

### Data collection

The managers of the isolation and quarantine facilities were asked to register the SARS-CoV-2 positive cases, within 24 h from the test result, in an online form of data collection specifically designed by INMP experts and available on a dedicated INMP electronic platform. The information flow collected, for each case of positivity found, information on the facility (name, location), on the case (age, sex, nationality), date of entry in Italy, date of entry in the facility, type of test performed to assess positivity, any symptoms and date of their onset; finally, any comorbidity and hospitalization.

SARS-CoV-2 positivity was defined according to the national guidelines then in force [9]. With regard to the observation period, a positive molecular test result for SARS-CoV-2 was initially required as proof of infection; subsequently, positivity by rapid antigen tests was also considered sufficient.

### Epidemiological measures and statistical analysis

Absolute frequencies and proportions (e.g. gender distribution) were used to describe qualitative variables. Mean and standard deviation (SD) were used to describe quantitative variables (e.g. age).

The following measures were analyzed: cumulative and monthly-stratified SARS-CoV-2 infection positivity rate - calculated as number of subjects with a positive swab out of all subjects tested-; cumulative and stratified by nationality SARS-CoV-2 infection incidence rate - calculated as number of positivities out of population at risk - (per 1,000) of newly arrived migrants, and characteristics of positive cases (sex, age, nationality, clinical presentation, comorbidities, hospitalization). 

The cumulative SARS-CoV-2 infection incidence rate in the resident population in Italy of the corresponding age group was also computed for the same observation period, based on the number of cases reported by the Italian Civil Protection Department [10] and the population resident in Italy according to the Italian National Institute of Statistics [11]. Incidence rate ratio (IRR) with 95% confidence intervals (95%CI) was calculated as ratio of the positivity rate among migrants with that of the resident population in Italy.

## Results

The land-based facilities and the nine ships used to carry out the isolation and quarantine of all newly arrived migrants transmitted their data into the information stream. Out of 70,512 migrants landed on the Italian shores during the observation period, our surveillance system has recorded a total of 2,861 positive cases (2,699 in 2021, 94 in 2022 and 19 unspecified in year) that were detected with the antigenic test in the 60.64% of cases (1,735) and with the molecular test in the remaining portion (1,126 cases).

### Cumulative and monthly-stratified SARS-CoV-2 infection positivity rates

A SARS-CoV-2 cumulative positivity rate of 4.06% was observed during the whole observation period.

**Fig. 1:**
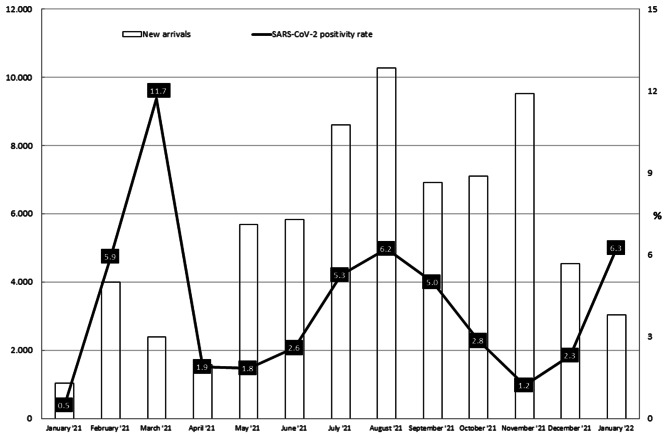
SARS-CoV-2 positivity rate in newly arrived migrants over the period of observation

Figure 1 shows the positivity rate observed over the months, and the amount of newly arrived migrants reported in the same period. A spike has been observed in March 2021 (positivity rate 11.73%) and an upward trend clearly emerges in late 2021. The low positivity rate observed in January 2021 (0.48% with only 5 cases recorded) suggests a potential under-notification of positive cases due to the recent launch of the flow.

### Cumulative incidence of SARS-CoV-2 infection in newly arrived migrants and in the resident population in Italy

As all newly arrived migrants have been tested, the positivity rate can be reasonably considered an accurate representation of the real incidence of cases in the observed population. Table 1 reports the cumulative incidence rate (per 1,000) of the SARS-CoV-2 infection in newly arrived migrants compared with that of the resident population in Italy of the same age group (< 60 years old) in the same period. Taking the SARS-CoV-2 incidence rate observed in the Italian resident population as a reference, the incidence rate ratio in newly arrived migrants is about three quarter lower (0.23) (Table 1).

**Table 1:** SARS-CoV-2 cumulative incidence rate for newly arrived migrants and resident population in Italy

	Population at risk	N. of cases	Cumulative incidence rate per 1.000 (95% CI)	Incidence rate ratio (95% CI)
**Newly arrived migrants**	70,512	2,861	40.6 (39.1–42.1)	0.23 (0.22–0.24)
Resident population*	40,902,006	7,265,656	177.6 (177.5-177.8)	Ref.

*<60 years old

CI: confidence interval

### Positive cases characteristics

The characteristics of the 2,861 positive cases are shown in Table 2.

**Table 2:** SARS-CoV-2 positive cases among newly arrived migrants: characteristics

Characteristics	N (%)
**Positive cases**	2,861
**Sex**	
Male	2,567 (89.72)
Female	294 (10.28)
**Age (Mean, SD)**	24.76 (7.62)
*Class age*	
0–4	32 (1.1)
5–9	33 (1.2)
10–14	56 (2.0)
15–19	533 (18.6)
20–24	898 (31.4)
25–29	664 (23.2)
30–34	342 (12.0)
35–39	182 (6.4)
40–44	75 (2.6)
45–49	35 (1.2)
50–59	11 (0.4)
**Nationality**	
*(5 most represented nationalities)*	
Tunisian	767 (26.81)
Egyptian	402 (14.05)
Bangladeshi	238 (8.32)
Ivorian	222 (7.76)
Iranian	128 (4.44)
Others	1,104 (38.59)
**Clinical presentation**	
Symptomatic	4 (0.14)
Asymptomatic	2,802 (99.86)

SD: standard deviation

Among positive cases, more than half (54.6%) belonged to the age group 20–29 and almost a quarter (654; 22.9%) were under the age of 18. No comorbidity has been reported and no cases have been hospitalized. In the 4 cases with symptomatology, body temperature of at least 37.5 °C was recorded, and other reported symptoms were fatigue, dry cough, vomiting, and sore throat.

### Focus on migrants’ nationality

Positive cases were mainly Tunisians and Egyptians. Indeed, they also represented the most frequent nationalities among newly arrived migrants in Italy during the period of observation (Table 3). SARS-CoV-2 incidence rates in newly arrived migrants stratified by the 5 most represented nationalities among the positive cases showed a higher incidence rate for Ivorian migrants, followed by Tunisian and Egyptian ones.

**Table 3:** Cumulative SARS-CoV-2 incidence rates in newly arrived migrants stratified by nationality

Nationality	Population at risk*	N. of cases	Incidence rate (per 1,000)
Tunisian	16,010	767	47.91
Egyptian	8,757	402	45.91
Bangladeshi	8,537	238	27.88
Ivorian	3,958	222	56.01
Iranian	3,915	128	32.70

* number of migrants of that nationality landed in Italy during the period of observation

## Discussion

Our information flow recorded all SARS-CoV-2 positivity detected in migrants who landed in Italy by sea between January 2021 and January 2022, showing a lower SARS-CoV-2 infection incidence rate compared to that of the resident population in Italy of the same age group, an asymptomatic presentation of the infection and a health profile characterized by the lack of comorbidity.

In particular, according to our study, the SARS-CoV-2 infection incidence in newly arrived migrants by sea in the observation period was less than a quarter of that found in the resident population of Italy.

Considering the systematic way in which the SARS-CoV-2 test is performed for all newly arrived migrants in Italy compared to the opportunistic screening performed in the Italian general population (in case of symptoms or contacts at high risk of positivity) it is reasonable to assume that the difference in the incidence of SARS-CoV-2 infection between the population resident in Italy and newly arrived migrants is, in reality, even greater than what our study shows, although the latter generally live in overcrowded conditions at departure and during the journey, conditions that can certainly represent an additional risk factor for the spread of the virus. Indeed, the literature argues that, in the case of non-systematic population screening, the effective SARS-CoV-2 infection is largely underreported, since asymptomatic cases often remain unnoticed and the asymptomatic clinical presentation represents over 70% of COVID-19 cases globally recorded in Italy since the beginning of the pandemic [12].

A study performed in 2020 on data from several European countries reported a ratio of total estimated cases/observed cases ranging from the 3.93 times in Norway to the 7.94 in France with Italy placed at 4.53 (bootstrap-based intervals: 4.51–4.58) [13], which means that for every observed positive case there are 3.5 infected persons unseen. Another study published in 2021 and fed with data up to June 2020 from three Italian regions highlighted a percentage of undetected infections of 52.4% (95% CI 52.2–52.6%) [14]. Unfortunately, the latest estimates are not available.

Several factors may contribute to the low SARS-CoV-2 infection rates observed in newly arrived migrants in Italy. Among these, the demographic factor could certainly play a role, particularly with regard to the countries of origin and transit of migrants landed in Italy.

Migrants arriving in Italy by sea are generally young adult males coming mainly from North Africa (mostly Tunisia and Egypt) and Asia (e.g. Bangladesh, Iran, Pakistan, Afghanistan, Iraq) and, basically, the COVID-19 pandemic did not change the demographic profile of newly arrived migrants.

Focusing on the main origin and transit countries of migrants reaching Italy by sea, the global COVID-19 incidence (since the beginning of the pandemic) is reported to be 8,830 cases per 100,000 in Tunisia; 8,612 in Iran; 1,186 in Bangladesh; 504 in Egypt, and 312 cases in Ivory Coast. These incidence rates are significantly lower than those reported in the European countries (e.g. 55,478 per 100,000 in Iceland, 48,335 in the Netherlands, 47,527 in Portugal or 45,263 in France, to mention a few) [1].

When considering the African region, many publications attempt to argue the reasons for the observed low burden of morbidity and mortality from COVID-19, also known as the “African paradox”. The young age of the population in sub-Saharan Africa, the lower rates of comorbidities and the potential cross-protection derived from previous exposure to circulating coronaviruses and other pathogens (including malaria parasites) are among the potential protective factors evoked against severe COVID-19 manifestations [15].

Regarding the low rates of SARS-CoV-2 infection, the most frequently mentioned theories are based on both environmental and biological factors. The lack of long-term care facilities [16] (where the virus spread especially in the first pandemic wave), the climate, [14] people’s tendency to spend time outdoors [17] but also genetic variants of the human angiotensin-converting enzyme-2 (ACE2) and the transmembrane serine protease 2 (TMPRSS2) receptors [18] and effective public health emergency mitigation strategies (already experienced during the Ebola outbreak) [16] were invoked to account for the phenomenon. Not least, a limited test capacity [19] was also claimed.

According to our study, since newly-arrived migrants are all tested for COVID-19 on arrival, reporting a significantly lower incidence rate than the resident population, if there is indeed a real underestimation of SARS - CoV-2 infection in countries of origin and transit this could not be the only explanation for the phenomenon. In this regard, it should be considered that the low rate of SARS CoV-2 infection on arrival may depend on the immunity acquired by migrants during the journey, as epidemiological studies indicate that people who have had COVID-19 previously are between 80% and 100% protected against reinfection [20]. Moreover, although COVID-19 is generally characterized by low case fatality rates (currently about 1.1% - world estimates - compared to the highest value of 7% globally observed in April-May 2020 [21]), it cannot be excluded that its severe symptomatology may inhibit migrants’ travel, thus leading - to some extent - to a sort of selection at the departure of individuals reaching host countries.

Interestingly, unlike the available statistics where Egypt and Ivory Coast report particularly low incidence rates of SARS -CoV-2, in our study the incidence rates observed in Egyptian and Ivorian migrants are roughly in line with those of other nationalities. Before the departure towards Italy, the stay of migrants in countries with higher SARS-CoV-2 incidence rates than the origin countries - for example Libya from which Egyptians and Ivorians also depart [22] - and the conditions of the journey itself (overcrowded boats) may offer a possible explanation for the phenomenon observed.

Regarding the clinical characteristics of the positive cases, no associated comorbidity was recorded. Although a possible explanation for this could lie in the initial medical assessment carried out at the border, which is often not oriented towards a thorough clinical evaluation of newly arrived subjects, this finding is in line with the low prevalence of comorbidities observed in other studies, [23] thus confirming a significant “healthy migrant effect” in migrants reaching Italy by sea.

The low rate of COVID-19 clinical manifestations observed upon arrival also deserves attention. The young age, which has been associated with higher asymptomatic rates of the SARS-CoV-2 infection, [24] and the widespread of variants more likely to cause asymptomaticity [25] may have played a role. However, recent studies have particularly highlighted higher rates of asymptomatic infections in Africa compared to other regions, [26] a finding that might imply the contribution of genetic and immunological factors.

### Strengths and limitations

To the best of our knowledge, our study represents the first observational example of systematic screening for COVID-19 in migrants newly arrived on European shores, providing solid information on the incidence of SARS-CoV-2 infection in migrants upon arrival. In this regard, the total coverage of the facilities and ships involved in the quarantine and isolation of newly arrived migrants bodes well for the robustness of the study data.

Focusing on possible study limitations, the study covers only migrants who reached Italy by sea, for whom reliable data are available from the Ministry of Interior’s accounting systems and the information flow managed by INMP. However, according to currently available estimates, the share of migrant flows entering Italy by land, is markedly lower (9,400 migrants arrived by land from Slovenia – the main point of entry in Italy for migrants crossing the Western Balkan Route – in 2021, according to UNHCR data). Nevertheless, since countries of origin and, especially, the conditions of the journey of migrants arriving in Italy by land are different from those of migrants reaching Italy by sea, our findings are not generalizable to this population group.

Further, since the participation in the information flow of facilities and ships, promoted and supported by the Italian Ministry of Interior, was not binding, we cannot exclude that some SARS-CoV-2 positive cases were not actually reported in the information flow. Finally, since newly arrived unaccompanied minor migrants are usually housed in separate facilities spread throughout Italy, our results cannot be extended to this specific sub-population.

## Conclusions

Our study shows a remarkable low rate of SARS-CoV-2 infection in migrants reaching Italy by sea compared to the Italian population. The implications of this finding are twofold: firstly, there is evidence that irregular migrants arriving in Italy during the observation period did not increase the burden of COVID-19 in the country; secondly, further studies are needed to investigate the possible reasons for the low incidence observed among migrants coming from countries mostly belonging to Africa and Asia. Furthermore, it is worth emphasizing that the realization and successful outcome of the study conducted are also due to the presence of a structured governance, both of the migrant reception system and of the prevention and management measures of COVID-19, including the provision of free health care to migrants in case of severe COVID-19. This underscores the importance for the health and migration policies of the various transit and destination countries to have reception systems with clear and structured governance as well as evidence-based national operational guidelines in order to protect individual and, consequently, collective health.

## Data Availability

All data relevant to the study are included in the article.

## Declarations

### Patient and public involvement

Patients and/or the public were not involved in the design, or conduct, or reporting, or dissemination plans of this research.

## Abbreviations

WHO: World Health Organisation
COVID-19: Coronavirus disease 2019
US: United States (of America)
INMP: Italian National Institute for Health, Migration, and Poverty
SARS-CoV-2: Severe acute respiratory syndrome coronavirus 2
SD: Standard deviation
IRR: Incidence rate ratio
CI: Confidence interval
ACE2: Angiotensin-converting enzyme-2
TMPRSS2: Transmembrane Serine Protease 2
